# Sleep quality and sleep routines as mediators of stressors and life satisfaction in Czech university students: a structural equation model

**DOI:** 10.3389/fpsyg.2023.1231773

**Published:** 2023-09-01

**Authors:** Michaela Prokeš

**Affiliations:** Department of Sociology, Faculty of Social Sciences, Charles University, Prague, Czechia

**Keywords:** stressors, sleep consistency, sleep quality, life satisfaction, university students, Czech Republic

## Abstract

**Introduction:**

Sleep is especially important to overall well-being. Some aspects of sleep have been well documented, for example sleep quantity and its effect on well-being, but the value of a consistent sleep routine remains poorly studied. University students are a population group especially susceptible to stress, mental health problems and poor sleep quality and experience changing daily schedules. Investigating the protective power of sleep in this population group is therefore an important avenue of research.

**Methods:**

Applying a structural equation model, the current study surveyed a large sample of Czech university students during the COVID-19 pandemic in late spring, 2021, and observed the mediation effects of sleep on this group.

**Results and Discussion:**

The study found that working, maintaining social contact and attending lectures in person had a strong effect on satisfaction with life. Increased personal study time indirectly supported consistent sleep routines and mediated perceptions of life satisfaction. As expected, the results indicated the importance of high-quality sleep. The results also verified partial mediation, directly and indirectly, through sleep quality, highlighting the significance of a consistent sleep routine in students on their self-reported satisfaction with life.

## Introduction

Emerging adulthood, the life stage between adolescence and adulthood typically ascribed to university students, opens up many opportunities for life exploration. But it also comes with instability (Arnett, 2015), and despite many positive rewards, the joy of student life can be outweighed by stressful experiences encountered during this period (Sreeramareddy et al., 2007; Leahy et al., 2010; Cvetkovski et al., 2012). For students, common stressors are academic and educational pressure (Stanley and Manthorpe, 2002; Monk, 2004; Cooke et al., 2006), relationship difficulties (Stanley and Manthorpe, 2002; Darling et al., 2007), and financial challenges (Stanley and Manthorpe, 2002; Monk, 2004). Extended working hours have also been linked to lower mental wellbeing (Ogawa et al., 2018; Wong et al., 2019). Evidence also suggests that students who spend more time studying alone report significantly more stress and less satisfaction with life (Coccia and Darling, 2016). It is not surprising, therefore, that this adjustment stage is considered one of the most stressful periods in life (Cress and Lampman, 2007; Chao, 2012).

Many researchers have reported findings that negative stresses generally cause a decrease in life satisfaction in students (Matheny et al., 2002; Barnes and Lightsey, 2005; Darling et al., 2007; Weinstein and Laverghetta, 2009; Abolghasemi and Taklavi Varaniyab, 2010; Schiffrin and Katherine Nelson, 2010; Lee et al., 2016; Puri et al., 2016). University students are a vulnerable group which experiences depressive symptoms (Mikolajczyk et al., 2008; Rückert, 2015), high levels of stress (Stanley and Manthorpe, 2002) and the highest prevalence of mental health problems than any other age group (Dahlin et al., 2005; Verger et al., 2010; Kumar, 2016). Despite the generally adverse impact of these stressors on life satisfaction, the link between life experiences during university studies and reported life satisfaction is also positive, stress buffers such as good academic experiences, living conditions (Chow, 2014) and social relationships (Mahmoud et al., 2012; Chow, 2014; Coccia and Darling, 2016; Kuang-Tsan and Fu-Yuan, 2017) being conducive to feelings of greater satisfaction with life (Chow, 2014).

Although the effects of various stressors on the overall wellbeing of students are thoroughly described and documented, the role of sleep’s protective power in this equation and age group is less well studied despite sleep being a key aspect of life. People oftentimes choose to cut back on sleep instead of other activities (Kroese et al., 2014) to gain more time during the day not realising the dire consequences such as elevated risk of depression (Al-Abri, 2015) and overall poorer health (Luyster et al., 2012). Yet, sleep is still lagging being considered a luxury instead of one of the pillars of a healthy lifestyle (Alvarez and Ayas, 2004). The existing evidence shows that higher stress levels lead to poorer sleep among medical students (Ahrberg et al., 2012; Almojali et al., 2017), nurses (Rocha and Figueiredo De Martino, 2010), police officers (Charles et al., 2011), patients with an elevated risk for cardiovascular disease (Kashani et al., 2012), but also adolescents (Yan et al., 2018) and university students during the COVID-19 pandemic (Benham, 2020). In addition to sleep quality, sleep routine is also likely to be affected by stress (Benham, 2020) as has been previously shown in students (Lund et al., 2010). Stress in general is a common source of impaired sleep (Kim and Dimsdale, 2007; Han et al., 2012). Healthy sleep routines and high-quality sleep are consistently linked to higher levels of wellbeing and life satisfaction in various population groups. In addition to students (Ness and Saksvik-Lehouillier., 2018; Shin and Kim, 2018), better sleep quality is also linked to greater life satisfaction in children (Blackwell et al., 2020) and older adults (Zhi et al., 2016; Duong, 2021; Papi and Cheraghi, 2021). Students, however, are more likely to have unhealthy sleep routines and frequent sleep disturbances (Mandelkorn et al., 2021), insufficient sleep duration (Lund et al., 2010; Gusman et al., 2021; Ulrich et al., 2021) and high levels of irregular sleep/wake routines (Lund et al., 2010).

Barber et al. (2010) suggested that sleep quality as a potential mediator for the relationship between stressors and sleep consistency could predict wellbeing in university students. This was partially demonstrated by Peach et al. (2016), who investigated the effect of sleep hygiene on depression and subjective wellbeing. However, these studies had significant limitations that the presented study aims to surpass: (1) both involved small samples (80 and 218 respondents), (2) neither acknowledged the effect of a broader range of stressors as independent variables, and (3) the study by Peach et al. (2016) applied a more universal sleep hygiene scale which consisted of only a few items for sleep routines. Duong (2021) also examined the effects and mediating roles of fear and anxiety in depression, sleep and wellbeing, focusing specifically on fear and anxiety associated with COVID-19 and the resulting sleep disturbances, yet (4) omitted more general routine stressors, sleep routines and life satisfaction from the research.

The need for scientific knowledge on the relationship between stress, sleep and life satisfaction in students became even more urgent during the COVID-19 pandemic: many of the above-mentioned stressors were amplified by COVID-19 emergency measures (e.g., university closures) and led to poorer mental wellbeing and satisfaction with life in people (Eurofound et al., 2021). A meta-analysis of 27 peer-reviewed studies from 15 countries indicated that in 2020, a larger than usual proportion of students presented with symptoms of anxiety, stress and depression (Batra et al., 2021). Other recent studies support this observation (Rogowska et al., 2020; Gusman et al., 2021; Ulrich et al., 2021). Satisfaction with life in students deteriorated (Rogowska et al., 2021). Almost 30% of students from 136 countries reported changes in their sleep routines (Ellakany et al., 2022). Some studies reported poorer sleep in students compared to the pre-pandemic period (Ulrich et al., 2021; Lukowski et al., 2022). Another study suggested that students did not experience any significant changes in sleep quality because the quality of sleep in the respondents was already very poor (Benham, 2020). This observation agreed with the results from other pre-pandemic studies on sleep quality in students (Yang et al., 2003; Oswalt and Wyatt, 2014). It is probable that sleep quality is also dependent on the context of when it is measured; for example, longitudinal studies show an initial increase in sleep quality during the initial COVID-19 outbreak but a continuous decrease over time as the pandemic progressed (Gusman et al., 2021).

Previous findings highlight the need for a deeper investigation of the relationship between consistent sleep routines, sleep quality and satisfaction with life reported by students. Although a wealth of previous research findings is available, few studies have considered sleep routines and sleep quality in relation to stressors and life satisfaction reported by students in pre-covid or covid pandemic contexts. The current study, therefore, analyses an extensive Czech student population sample of 2,488 respondents during the third COVID-19 wave in 2021. The sample group was likely to have been vulnerable to feelings of compromised wellbeing during this period and provided a useful basis for the study of the relationship between various stressors, life satisfaction and routines mediated by sleep quality.

## Hypotheses

The existing literature suggests a negative correlation between high-stress load and life satisfaction in Czech students. Specifically, in the proposed model offline course load, online study, personal study time, time spent in pad work and decrease in social contact due to COVID-19 measures are predictors of life satisfaction and two sleep variables, sleep routines and sleep quality, are incorporated into the model as potential mediators for the association between the five mentioned stressors and life satisfaction. The current study hypothesises that a high frequency of online classes, not attending any classes in person, long hours of personal study and work, and a decrease in social contact during the pandemic period are associated with inconsistent sleep routines and lower life satisfaction directly, and with poor sleep quality indirectly through sleep routines (H1). Sleep routines are expected to serve as a buffer in the relationship between stressors and life satisfaction (H2). It is also expected that irregular sleep routines and poor-quality sleep are independently linked to lower life satisfaction (H3) and that their effects on life satisfaction remain consistent if sleep quality is incorporated into the model as a mediator for the association between stressors, sleep routine and life satisfaction (H4).

## Data and methods

### Description of the data set

**Figure 1:**
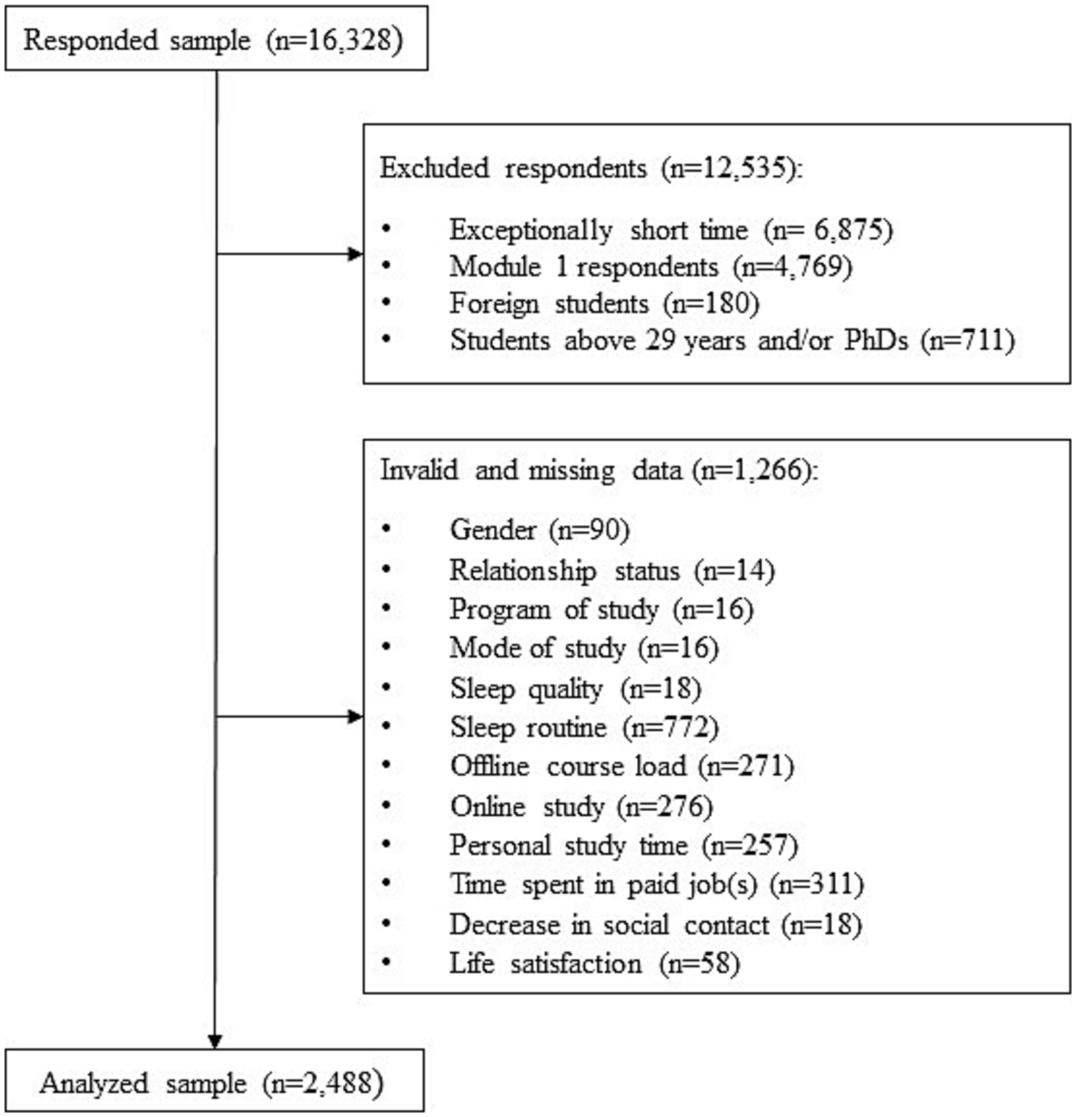
Study process scheme.

The cross-sectional data collection survey, whose data is used in the current study, was executed during the end of the third COVID-19 wave in the Czech Republic (18.05.2021–30.06.2021). The survey was conducted online by the Faculty of Social Sciences of Charles University and the Institute of Sociology of the Czech Academy of Sciences, focusing on students enrolled in university study in the Czech Republic and the effects of the pandemic on their studies, feelings of wellbeing and other related topics. All public and private Czech Universities were contacted and asked to participate in the survey by sending an email invitation and link to the questionnaire to their students. In total, 16,328 student respondents from more than 23 universities responded to the questionnaire. After the data collection was complete, the data was checked for the allowed range of values of categorical and numerical variables and checking the consistency of the data against the filtering conditions. Logic check and cleaning were done. Respondents with exceptionally short time were excluded from the data set. The questionnaire was extensive (110 questions) and designed around a core of basic questions on sociodemographic background, studies and wellbeing, plus two additional modules, one which contained psychometric scales and questions about physical activity and the other about romantic relationships and sleep. All students completed the core questionnaire and were then randomly redirected to one of the two additional modules. This significantly reduced the time required to complete the survey, but it also decreased the size of the sample for the modules by approximately half. Only Czech students below the age of 29 were considered for the analysis. Doctoral students were omitted from the survey because their research routines more resemble employment than undergraduate studies. The final sample for analysis in this article was 2,488 students (see Figure 1 for details).

### Measured parameters

#### Dependent variable

Life satisfaction was measured according to a five-item Satisfaction with Life Scale (SWLS) (Diener et al., 1985). The respondents indicated their agreement on a scale of 1 (strongly disagree) to 7 (strongly agree) with the five given statements. The statements were as follows: *In most ways, my life is close to ideal* / *The conditions of my life are excellent* / *I am satisfied with my life*/*So far, I have the most important things I want in life* / *If I could live my life over, I would change almost nothing*. The resultant reliability estimates were excellent (*α* = 0.879). SWLS is a proven, valid and reliable measurement tool for use with diverse population groups (Pavot and Diener, 2009).

#### Mediating variables

The current study measured sleep quality according to the question: *How would you rate the quality of your sleep?* on a four-point Likert scale with responses *very good/good/bad/very bad*. Other studies have also used this parameter to measure sleep quality (Shao et al., 2010; Ness and Saksvik-Lehouillier., 2018). Data on sleep were collected using the Munich Chronotype Questionnaire (MCTQ) (WEP, 2020). However, no unified methodology currently exists for measuring sleep consistency. A very common measurement parameter is social jetlag, which describes the misalignment between biological and social preferences, typically calculated as the mid-sleep difference in time between workdays and free days (Jankowski, 2015). This procedure requires that respondents report the time of sleep onset and sleep end every day. The resulting sleep durations for workdays and free days are then calculated and compared. This parameter, however, measures only the difference between workdays and free days, and does not consider that sleep routines may still be somewhat regular. The current study also asked students four MCTQ questions about sleep on workdays and free days (onset and end) but included the options to respond with cannot say/completely irregularly. The aim, however, was to measure students’ sleep routines and to calculate the consistency of their sleep habits. To address this aim, the study distinguished between students for whom it was possible to calculate sleep duration denoted as having *regular sleep* and hence value one, students with *regular sleep and suffering from social jetlag (value two)* and students who indicated irregular workdays, free days or completely *irregular sleep routines* (with value three). The main criteria, therefore, was the consistency of the routine and not the specific time of sleep onset or sleep end.

#### Independent variables

Study load was measured with three complementary variables: offline course load (dummy variable to determine whether offline courses were held), online study (teaching materials delivered through online courses such as lectures, seminars, labs, tests held online) and personal study time (preparation, learning, reading, homework, etc.). The variables for online study and personal study time were measured with four scales: 0 h, 1–10 h, 11–20 h, 21 or more hours. Time spent in paid work (not including student jobs during vacations) was measured in the same manner. All the above-mentioned stressors referred to the student’s prior week before filling in the survey.

Finally, decrease in social contact due to COVID-19 measures was calculated from a question which investigated the frequency of social contact: *“Did you have more or less contact (offline and online combined) with friends since the implementation of the first COVID-19 measures?”* The resulting dummy variable had two categories, *no positive change* indicated with “0” and *yes* indicated with “1.”

#### Covariates

The control variables used in the analysis were age (minimum age 17 years, maximum age 29 years), gender (male, female), study programme (bachelor’s, master’s), relationship status (*Are you currently in a steady relationship?*, with the option to answer *yes* or *no*), and study mode (on-site or combined).^1^

**Table 1:** Variables in the analytical sample of Czech university students.

		n (%)	Mean (SD)
Age			22.8 (2.0)
Gender			
	Male	938 (37.7)	
	Female	1,550 (62.3)	
Relationship status			
	Single	1,136 (45.7)	
	Steady relationship	1,352 (54.3)	
Study programme			
	Bachelor	1,596 (64.2)	
	Master	892 (35.9)	
Study mode			
	On-site	2,317 (93.1)	
	Combined	171 (6.9)	
Sleep quality			2.1 (0.8)
Sleep routine			
	Regular	392 (15.7)	
	Regular with social jetlag	1,502 (60.4)	
	Irregular	594 (23.9)	
Offline course load			
	None	1,923 (77.3)	
	Offline courses taking place	565 (22.7)	
Online study			
	None	653 (26.3)	
	1–10 h per week	870 (35.0)	
	11–20 h per week	648 (26.1)	
	21 and more hours per week	317 (12.7)	
Personal study time			
	None	121 (4.9)	
	1–10 h per week	1,083 (43.5)	
	11–20 h per week	639 (25.7)	
	21 and more hours per week	645 (25.9)	
Time spent in paid work			
	None	1,240 (49.8)	
	1–10 h per week	527 (21.2)	
	11–20 h per week	383 (15.4)	
	21 and more hours per week	338 (13.6)	
Decrease in social contact due to COVID-19 measures			
	No/positive change	850 (34.2)	
	Yes	1.638 (65.8)	
Life satisfaction			4.3 (1.3)

University students during COVID-19 pandemic, 2021 (*N* = 2,488).

### Analytical strategy

To examine the role of sleep quality, the study used Stata 17.0 statistical software. A structural equation model (SEM) estimated using Maximum Likelihood with robust standard errors (MLR) was applied to perform mediation analysis, and the software’s *medsem* package (Mehmetoglu, 2018) provided post-estimation results. ML is the most common estimation method especially suitable for large samples (Gutterres et al., 2012). It is ideally used in structural models with continuous outcomes (Rhemtulla et al., 2012; Finney et al., 2016) and outcomes with several categories (Rhemtulla et al., 2012) with three being the least needed to obtain valid and reliable results (Robitzsch, 2020). Adjustments on standard errors such as the applied robust standard errors approach are generally recommended to correct for possible bias using categorical outcomes in SEM (Gutterres et al., 2012). In addition, the *medsem* package performs a bias-correcting Monte Carlo test which is less consuming than the bootstrap test but still acceptable (Jose, 2013).

Widely used and recommended post-estimation tests for the overall goodness of fit were also performed to obtain a comparative fit index (CFI), root mean square error of approximation (RMSEA), Bayesian information criterion, Tucker Lewis index (TLI) and standardised Root Mean Square Residual (SRMR) (Hu and Bentler, 1999). These techniques enabled the generation of the separate direct, indirect and total effects of stressors, sleep quality and sleep routines on life satisfaction in Czech students. Ineligible respondents and invalid data were excluded prior to the analysis (see Figure 1) and therefore there were no missing data for any of the variables included into the analysis. Demographics were included in the model as covariates. Relationships between variables were tested before analysis (see correlation matrix in Supplementary material 1). The resultant model fit demonstrating the adequacy of the proposed model was acceptable in all test criteria except for chi-square which was significant at *p* < 0.01 [*χ*^2^ = 20.762 (5df). *p* = 0.001]. However, chi-square statistics is common to be sensitive to large sample sizes (Bentler and Bonett, 1980; Jöreskog and Sörbom, 1993). Other model fit statistics used to evaluate the presented model were RMSEA (0.036) and SRMR (0.010) which are in combination recommended by Hu and Bentler (1999) to range from zero and 0.060 for RMSEA and zero to 0.090 for SRMR. The resultant TLI (0.857) close to 0.90 or 0.95 generally reflects a good model fit (Schumacker and Lomax, 2016). As for the CFI (0.978), a value close to 1.0 is recognised as an indication of a good fit (Hu and Bentler, 1999). Model effects were estimated using standardised path coefficients with 95% confidence intervals (CIs, two-tailed) and test statistics. Effect sizes of the dependent variables were estimated (R^2^_LFSAT_ = 0.220, R^2^_SLEQUAL_ = 0.028, R^2^_SROUTINE_ = 0.012) using standard R^2^ measure (Bentler and Raykov, 2000).

**Figure 2:**
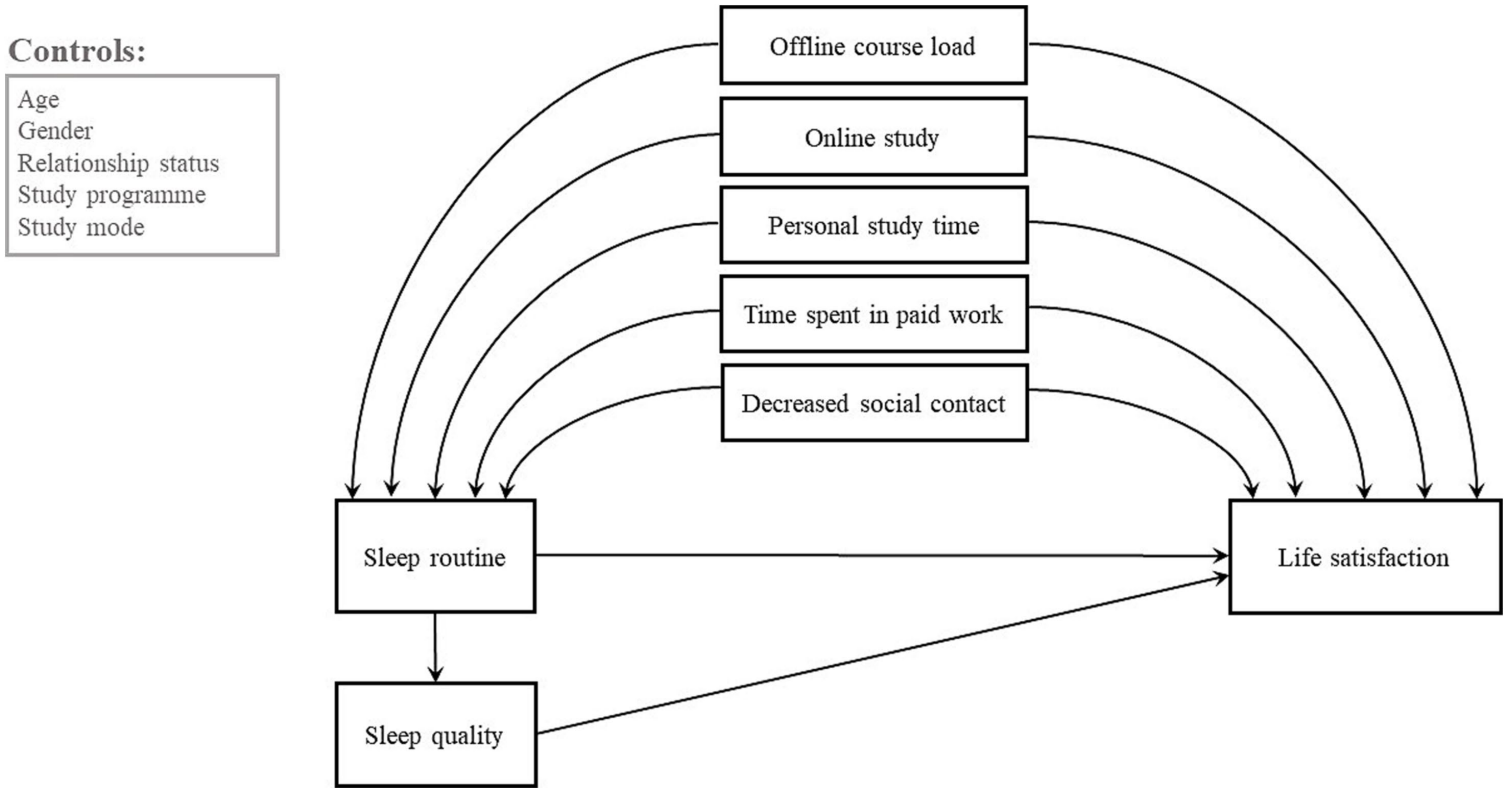
Conceptual model of the relationship between stressors, sleep and life satisfaction.

The conceptual model is illustrated in Figure 2. Based on the literature review, the model assumes a direct effect of stressors, sleep quality and sleep routines on life satisfaction in Czech students and an indirect effect of parallel mediators sleep routines and sleep quality as a mediator while also considering some basic control variables.

## Results

Table 1 indicates that the Czech student sample (*N* = 2,488) used for analysis was predominantly female (62.2%). Although more of the sampled students reported being in a steady relationship (54.3%) than not, both categories were roughly balanced. Given the low age average (22.8), it is not surprising that the majority were students at the bachelor’s degree level (64.2%), and only about a third (35.9%) of the sample were at a master’s degree level. The overwhelming majority of students were in regular full-time on-site study (93.1%). Students overall reported rather good sleep quality (mean score 2.1); almost one-fourth of them (23.9%) were classifiable as completely irregular sleepers while about two-thirds (60.4%) of students fall in the category of regular sleepers but suffering from some level of social jetlag. As for their study load, about one-fourth of students (22.7%) reported having at least some offline courses towards the end of the third wave of the pandemic in the Czech Republic. Instead of usual offline courses, online courses were taking place, most commonly between 1 and 10 h per week (35.0%). However, it was no exception that a portion of students had no online classes (26.3%). Personal study was the most frequent substitute for any other type of study and it also meant the heaviest load for students: about four in 10 (43.5%) had between 1 and 10 h per week and more than a half (50.6%) engaged in 11 or more hours of self-study per week. About half of the student sample were unemployed (49.8%), having no job or part-time job at the time of the data collection. Unsurprisingly, about two-thirds (65.8%) admitted a decrease in social contact with friends and family. And yet, the students tended towards being satisfied with their lives (mean score 4.3).

**Figure 3:**
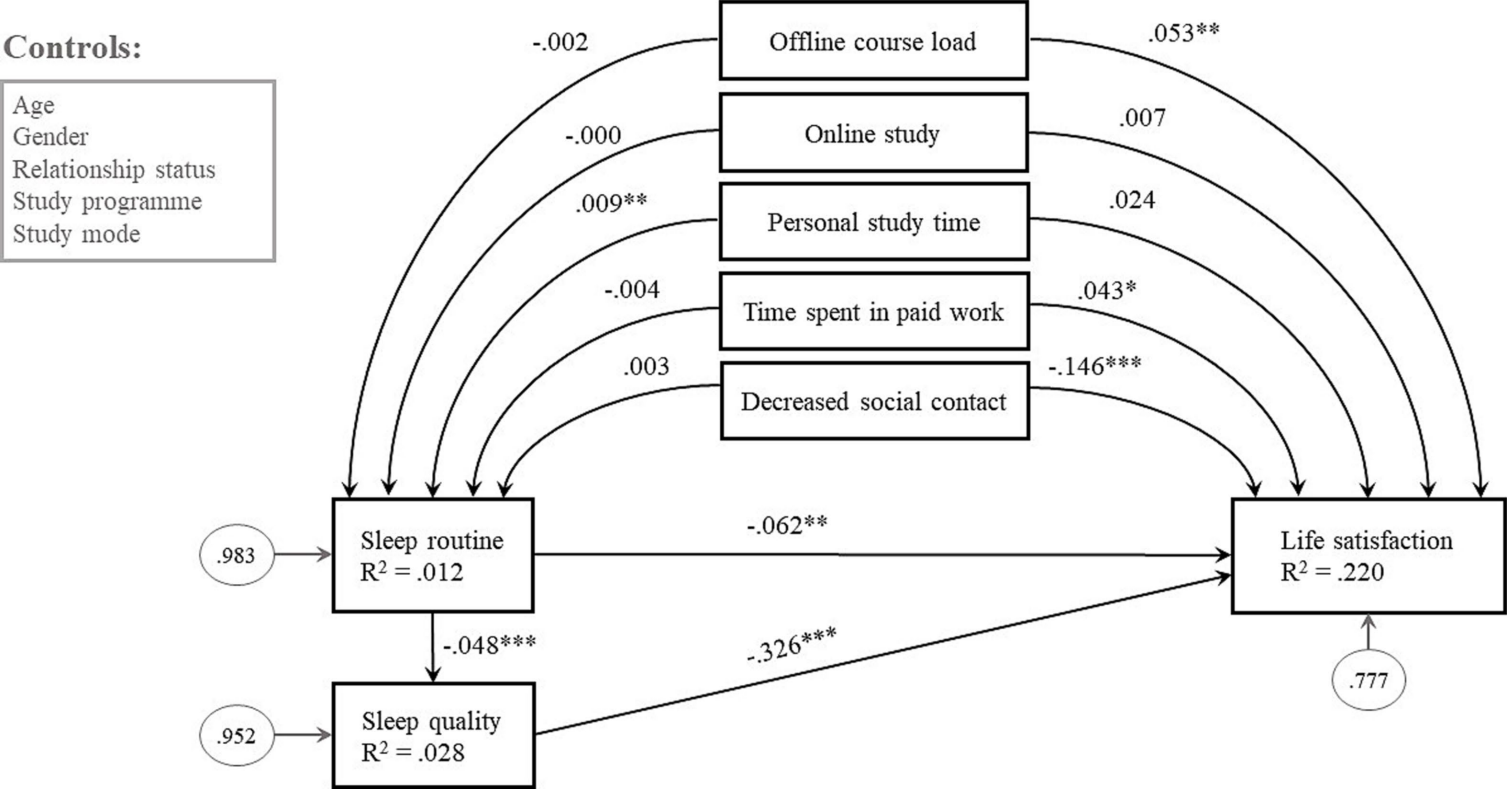
Standardised regression effects in structural model exploring the effect of stressors and sleep on life satisfaction. *N* = 2,488. *p < 0.05, **p < 0.01, ***p < 0.001. Model fit statistics: χ^2^ = 20.762 (5df). *p* = 0.001. CFI = 0.978. TLI = 0.857. RMSEA = 0.036. SRMR = 0.010. BIC = 88,334.112.

The results only partially supported the hypothesis (H1) that stressors are directly linked to sleep routines and life satisfaction and indirectly to sleep quality (Figure 3). From the measured academic stressors (various types of study load), participation in offline courses suggested a positive link to greater life satisfaction (*β* = 0.053; *p* < 0.01). Similarly, time spent working (*β* = 0.043; *p* < 0.05) affected satisfaction with life. The analysis revealed that students who experienced diminished social contact with friends during the COVID-19 pandemic outbreak also experienced decreased satisfaction with life (*β* = −0.146; *p* < 0.001). Online study (*β* = 0.007) and personal study time (*β* = 0.024) had no effect. As for the direct effect of stressors on sleep routine and indirect effect on sleep quality, only personal study time was significant (*β_direct_* = −0.082; *p* < 0.001; *β_indirect_* = −0.012; *p* < 0.01; see Supplementary materials 2, 3). Examination of the relationship between stressors and sleep routines (H2) revealed that in-person lectures (*β* = −0.002), the frequency of online classes (*β* = −0.000), time spent working (*β* = −0.004) and decreased social contact (*β* = 0.003) had no direct effect on sleep routine or indirect effect on life satisfaction. The effect of personal study time on sleep routines was significant (*β* = 0.009; *p* < 0.01). Moreover, full mediation manifested through the sleep routines (17.4% of the effect of personal study on life satisfaction is mediated by sleep patterns).

Analysis of sleep variables indicated that both sleep quality and sleep routines were significant, as hypothesised (H3). Poor quality sleep (*β* = −0.326; *p* < 0.001) and inconsistent sleep routines (*β* = −0.062; *p* < 0.01) had strong direct negative effects on overall life satisfaction. The effect of sleep routine was partially mediated by sleep quality (*β_indirect_* = −0.048; *p* < 0.001). Incorporating the effects of mediators (H4), the results indicated that 43.7% of the effects of sleep routines on life satisfaction were mediated by sleep quality. The mediation effect was also approximately 0.8 times greater than the direct effect of sleep routines on life satisfaction.

From the control variables for life satisfaction referred to as direct effects in Table 2, only gender suggested no effect (*β* = −0.010). Study mode was significant at a 99% level (*β* = −0.060), suggesting that combined study slightly lowered satisfaction with life. The strongest predictor was relationship status, indicating a link between being in a steady relationship and greater life satisfaction (*β* = 0.227; *p* < 0.001). Younger (*β* = −0.152; *p* < 0.001) and bachelor’s degree students (*β* = −0.104; *p* < 0.001) also tended to be more satisfied with their lives than older students and students enrolled in master’s degree studies.

**Table 2:** Standardised effects for predictors in the structural model determining life satisfaction among Czech students.

	Std. coef.	*p*	95% CI		Upper	Lower
Direct effects				
Age → LFSAT	−0.152	<0.001	−0.199	−0.104
Gender → LFSAT	−0.010	0.594	−0.045	0.026
Relationship status → LFSAT	0.227	<0.001	0.194	0.265
Program of study → LFSAT	−0.104	<0.001	−0.142	−0.065
Mode of study → LFSAT	−0.060	0.003	−0.101	−0.020
Sleep quality → LFSAT	−0.326	<0.001	−0.363	−0.289
Sleep routine → LFSAT	−0.062	0.001	−0.099	−0.025
Offline course load → LFSAT	0.053	0.003	0.018	0.087
Online study → LFSAT	0.007	0.715	−0.029	0.042
Personal study time → LFSAT	0.024	0.200	−0.029	0.042
Time spent in paid job(s) → LFSAT	0.043	0.028	0.005	0.082
Decrease in social contact due to COVID-19 measures → LFSAT	−0.146	<0.001	−0.180	−0.112
Indirect effects				
Sleep routine → SLEQUAL → LFSAT	−0.048	<0.001	−0.063	−0.034
Offline course load → SROUTINE → LFSAT	−0.002	0.459	−0.006	0.003
Online study → SROUTINE → LFSAT	−0.000	0.956	−0.004	0.004
Personal study time → SROUTINE → LFSAT	0.009	0.002	0.003	0.015
Time spent in paid job(s) → SROUTINE → LFSAT	−0.004	0.148	−0.009	0.001
Decrease in social contact due to COVID-19 measures → SROUTINE → LFSAT	0.003	0.166	−0.001	0.008
Total effects				
Sleep routine → LFSAT	−0.110	<0.001	−0.149	−0.072
Offline course load → LFSAT	0.051	0.004	0.016	0.086
Online study → LFSAT	0.006	0.722	−0.029	0.042
Personal study time → LFSAT	0.033	0.079	−0.004	0.070
Time spent in paid job(s) → LFSAT	0.040	0.045	0.001	0.079
Decrease in social contact due to COVID-19 measures → LFSAT	−0.143	<0.001	−0.177	−0.109

*N* = 2,488.

*p < 0.05, **p < 0.01, ***p < 0.001.

Model fit statistics: χ^2^ = 20.762 (5df). *p* = 0.001. CFI = 0.978. TLI = 0.857. RMSEA = 0.036. SRMR = 0.010. BIC = 88,334.112.

Effect size values: R^2^_LFSAT_ = 0.220, R^2^_SLEQUAL_ = 0.028, R^2^_SROUTINE_ = 0.012.

LFSAT, life satisfaction; SLEQUAL, sleep quality; SROUTINE, sleep routine.

## Discussion

The current study extends the existing knowledge on the relationship between stressors, sleep and wellbeing of Czech university students. The key findings highlight the relevance of personal social contact in various forms and the importance of sleep consistency and high sleep quality for feelings of satisfaction with life in this population during the waning days of the COVID-19 pandemic.

The results for hypothesis H1 deliver two important findings about the effect of stressors on life satisfaction. The first finding is that the effect of online and study load on life satisfaction appears to be negligible. This is in direct contrast to other studies which have suggested that academic load (Stanley and Manthorpe, 2002; Monk, 2004; Cooke et al., 2006) has a significant effect; the hypothesis (H1) was therefore not verified by the collected data. The pandemic context may, however, provide an explanation: activities which are performed daily or weekly, i.e., routines which provide structure throughout the day, are generally considered beneficial to individual wellbeing (Arlinghaus and Johnston, 2019). Therefore, while academic load could have been significant stressor in the non-pandemic times, its negative effect was instead balanced by the benefit of a structured day. This is also a possible explanation as to why a higher workload was revealed to have a positive effect on life satisfaction instead of serving as a stressor as was previously suggested by the literature (Ogawa et al., 2018; Wong et al., 2019). The second finding is that a decrease in social contact is a significant negative stressor, i.e., staying in touch with friends and attending in-person lectures and seminars has important benefits. It is probable that the pandemic and emergency restrictions diminished feelings of wellbeing and life satisfaction in the first place and may therefore have been the reason for these two variables having the strongest effect of all the measured stressors. Positive social relations are also consistently cited as beneficial and increasing life satisfaction (Mahmoud et al., 2012; Chow, 2014; Coccia and Darling, 2016; Kuang-Tsan and Fu-Yuan, 2017). Other studies conducted during the pandemic have also concluded that students prefer in-person classes to online learning, mainly because online mode involves adversely-perceived behaviours such as social distancing and produces feelings of isolation (Kanojiya, 2020; Lemay et al., 2021). As for the link between stressors and sleep, the solely increased load of self-study negatively affected the quality of sleep to a small extent. It also led to a more regular sleep routine, suggesting that more academic responsibilities tend to force the students to structure their days to meet all the course requirements. Yet, the negligible effect of the majority of included stressors is surprising given the fact that most of the literature suggests that stress, in general, is associated with poor sleep (Rocha and Figueiredo De Martino, 2010; Charles et al., 2011; Ahrberg et al., 2012; Kashani et al., 2012; Almojali et al., 2017; Yan et al., 2018) during standard non-pandemic times as well as during the pandemic (Benham, 2020), and also inconsistent sleep (Lund et al., 2010; Benham, 2020). The possible explanation as to why the chosen stressors had no effect, could be that students had other worries that were more pressing in their situation such as financial difficulties. Or maybe the students’ sleep is more likely to be affected by psychological problems, especially in the COVID-19 times even though already fading.

Even though it was expected that consistent sleep routines would serve as a buffer in the link between stressors and life satisfaction (H2), the results do not fully support this assumption. Only one stressor, personal study time, was indirectly associated with life satisfaction and fully mediated by sleep patterns suggesting that increased time spent by self-study is linked to more irregular sleep routines and subsequently lowering life satisfaction. Although this finding hints towards a consistent sleep routine being a potential buffer, the effect is fairly small and there is likely to be an effect on one or more other latent variables which were not included in the model.

The study corroborated the hypothesis (H3) that sleep and life satisfaction in Czech students are linked. The results of the analysis of the effect of sleep quality agreed with a number of previous studies on various populations (Ness and Saksvik-Lehouillier., 2018; Shin and Kim, 2018; Blackwell et al., 2020; Duong, 2021; Papi and Cheraghi, 2021). The results also showed that sleep routines have a significant effect on life satisfaction, supporting examples in the literature which have already alluded to this link to overall wellbeing (Bates et al., 2002; Fuligni and Hardway, 2006; Chaput et al., 2020) despite insufficient knowledge on this mechanism. The question, however, is whether it is a regular sleep routine which benefits students or whether the regular sleep routine is the result of a generally healthy lifestyle or certain personal traits that make the difference between the students with consistent and inconsistent sleep routines. Future studies can address these uncertainties.

The hypothesis (H4) regarding partial mediation also cannot be rejected. Analysis indicated that the effect of sleep routines not only remained consistent but was magnified to a great extent. This observation extends the knowledge of a study which implied that sleep quality could play an important role as a mediator between sleep consistency and student wellbeing (Barber et al., 2010) and verifies a previously conducted small-scale study (Peach et al., 2016), even though the author applied a more universal sleep hygiene scale and not sleep consistency specifically in that study. This is an important finding which extends the limited knowledge in the literature on the protective role of sleep, especially sleep routines which are yet to be explored further, preferably in a general population during more standard times. The presented study, however, implies that sleep routines are potentially very important yet to this day omitted from most of the stress, sleep and also wellbeing-related literature.

### Study limitations

The current study contributes an important discussion on the protective power of sleep and its link to general satisfaction with life, and although it has many advantages, introduces novel ideas and provides an extensive sample analysis, it also has some major drawbacks. The study provides valuable insight into student life, but unfortunately refers only to the Czech population and does not provide any international comparison. The study’s cross-sectional design is a limitation which does not permit a comparison with the pre-pandemic and post-pandemic periods. Sleep determinants were measured only cross-sectionally in the period shortly before summer, 2021. A longitudinal study would provide a further assessment of students’ sleep routines and feelings of wellbeing. In addition, no conclusion on the causal effects is possible since the study is correlational: whether in/consistent sleep routines are a cause or the result of decreased or increased wellbeing is yet to be clarified. Although some longitudinal studies (Totterdell et al., 1994; Paunio et al., 2009) have suggested that sleep quality affects feelings of life satisfaction, no evidence exists for the reverse relationship. It is more likely therefore that the results in the current study reflect sleep as a factor which affects life satisfaction rather than the reverse.

Ideally, sleep should not be self-reported and be measured objectively instead using devices such as smartwatches. Although studies have reported reasonable agreement between self-reporting and objective methods of measuring sleep (Gangwisch et al., 2006), the findings must still be interpreted with caution. Undeniably, the questionnaire may not have captured the influence of other variables; for example, daytime naps or parenthood may be factors in reported sleep quality and feelings of life satisfaction. Finally, the current study is contextual: sleep habits and routines were captured during the transition from lockdown to eased restrictions, but universities were largely closed and restricted throughout the entire data collection period, which coincided with the exam period starting in spring, 2021. The reality, therefore, might be different during extended breaks, vacations, or periods of self-isolation under the most restrictive regimes; for example, one study found that the midpoint of sleep for school children was later by an average of 1.5 h during the summer break (Moreno et al., 2021). The academic year for university students has a similar schedule, usually switching from classes to exams in June and July; this could also have affected the results.

## Conclusion

The aim of the study was to measure life satisfaction reported by Czech university students in relation to social contact, chosen academic and work stressors, and sleep. The study’s hypotheses predicted that heavy study loads and workloads, insufficient social contact, poor sleep routines and lack of quality sleep would adversely impact overall feelings of satisfaction with life. The indirect effect of sleep routines through sleep quality as partially suggested in past studies was also expected. Based on these hypotheses, the study applied a hypothetical model which uniquely combined stressors and sleep variables as predictors of life satisfaction in a vulnerable population group of around two and a half thousand students during the period which extended into the third wave of the COVID-19 pandemic in the Czech Republic.

The results delivered some interesting findings. Extensive study hours did not contribute to life satisfaction, whereas workload, social contact and in-person lectures proved to be significant positive stressors on the perception of life satisfaction in students. More hours spent by personal study, however, were beneficial to maintaining a consistent sleep routine but also causing poorer sleep. In relation to this finding, increased personal study time also had a small indirect positive effect on feelings of life satisfaction by promoting consistent sleep routines also as a mediator between personal study and life satisfaction. All of these findings are probably amplified by the context of the pandemic at the time of data collection. Good quality sleep especially and a well-structured sleep routine (both directly and indirectly) substantially raise satisfaction with life.

Sleep is undeniably important, yet widely underestimated. Much has already been discovered about sleep, but the power of a sleep routine as a potential protective factor in life is yet to be determined. Although the presented study contributes to the existing literature by highlighting the potential importance of sleep quality and the relatively novel concept of sleep consistency for future investigations, mechanisms involving stressors and buffers remain understudied in this concept. Challenges for future research include obtaining a longitudinal multi-country dataset which is representative of the student population and extendable to the general population.

## Data availability statement

The datasets presented in this study can be found in online repositories. The names of the repository/repositories and accession number(s) can be found at: https://doi.org/10.14473/CSDA/NCKCXO, Czech Social Science Data Archive (CSDA).

## Ethics statement

The studies involving humans were approved by Research Ethics Committee of the Faculty of Social Sciences, Charles University in Prague. The studies were conducted in accordance with the local legislation and institutional requirements. The participants provided their written informed consent to participate in this study.

## Author contributions

MP: study conception and design, data collection, analysis and interpretation of results, and manuscript preparation.

## Funding

This study was supported by the Charles University (project GA UK No. 160223).

## Conflict of interest

The author declares that the research was conducted in the absence of any commercial or financial relationships that could be construed as a potential conflict of interest.

## Publisher’s note

All claims expressed in this article are solely those of the authors and do not necessarily represent those of their affiliated organizations, or those of the publisher, the editors and the reviewers. Any product that may be evaluated in this article, or claim that may be made by its manufacturer, is not guaranteed or endorsed by the publisher.

## Footnotes

^1^Combined study is a combination of distance and full-time study. It is more demanding in terms of accommodation and requires greater student independence. The most notable difference from on-site study is that students spend significantly less time on campus and usually meet others on weekends (e.g., every second weekend). This study mode is especially suitable for individuals who want to complete their tertiary education while gainfully employed.
