# Supplementary material for: Sleep quality and sleep routines as mediators of stressors and life satisfaction in Czech university students: a structural equation model
Source: Front Psychol. 2023 Sep 1;14:1231773. doi: 10.3389/fpsyg.2023.1231773 (PMC10505808; doi:10.3389/fpsyg.2023.1231773)
Supplement: Supplementary file 1 [file Data_Sheet_1.docx]

Supplementary Material

Sleep Quality and Sleep Routines as Mediators of Stressors and Life Satisfaction in Czech University Students: A Structural Equation Model

Mgr. Michaela Kudrnáčová MSSc.*

*** Correspondence:** Corresponding Author: [40672727@fsv.cuni.cz](mailto:40672727@fsv.cuni.cz)

| **Table SM1. Correlation matrix of variables used in structural equation model** | | | | | | | | | | | | | | | | | | |
| --- | --- | --- | --- | --- | --- | --- | --- | --- | --- | --- | --- | --- | --- | --- | --- | --- | --- | --- |
| *Variables* | | Age | Gender | Relationship status | Program of study | Mode of study | Sleep quality | Sleep routine | | Offline course load | | Online study | | Personal study time | | Time spent in paid job(s) | Decrease in social contact | Life satisfaction |
| Age | | 1.000 |  |  |  |  |  |  | |  | |  | |  | |  |  |  |
| Gender | | 0.023 | 1.000 |  |  |  |  |  | |  | |  | |  | |  |  |  |
| Relationship status | | 0.132*** | 0.203*** | 1.000 |  |  |  |  | |  | |  | |  | |  |  |  |
| Program of study | | -0.425*** | -0.023 | -0.063*** | 1.000 |  |  |  | |  | |  | |  | |  |  |  |
| Mode of study | | -0.314*** | -0.042** | -0.098*** | -0.012 | 1.000 |  |  | |  | |  | |  | |  |  |  |
| Sleep quality | | 0.002 | 0.010 | -0.049*** | 0.048*** | 0.003 | 1.000 |  | |  | |  | |  | |  |  |  |
| Sleep routine | | -0.016 | 0.005 | -0.023 | 0.024 | -0.026 | 0.147*** | 1.000 | |  | |  | |  | |  |  |  |
| Offline course load | | -0.091*** | 0.028* | -0.014 | -0.076*** | 0.042** | -0.014 | 0.013 | | 1.000 | |  | |  | |  |  |  |
| Online study | | -0.209*** | -0.028* | -0.033* | 0.075*** | 0.125*** | -0.005 | 0.000 | | 0.004 | | 1.000 | |  | |  |  |  |
| Personal study time | | -0.057*** | 0.011 | -0.040** | -0.055*** | 0.088*** | 0.023 | -0.087*** | | 0.056*** | | 0.045*** | | 1.000 | |  |  |  |
| Time spent in paid job(s) | | 0.302*** | 0.050*** | 0.131*** | -0.112*** | -0.257*** | -0.028* | 0.045** | | -0.064*** | | -0.099*** | | -0.169*** | | 1.000 |  |  |
| Decrease in social contact | | -0.021 | 0.001 | 0.012 | 0.033* | 0.049*** | 0.065*** | -0.050*** | | -0.037** | | 0.043** | | 0.074*** | | -0.071*** | 1.000 |  |
| Life satisfaction | | -0.001 | 0.031* | 0.231*** | -0.091*** | -0.032** | -0.368*** | -0.107*** | | 0.047*** | | -0.005 | | -0.016 | | 0.072*** | -0.149*** | 1.000 |
| **** p<0.01, ** p<0.05, * p<0.1* | | | | | | | | | | | | | | | | | | |
| **Table SM2. Standardized effects for predictors in the structural model determining life satisfaction among Czech students: sleep patterns** | | | | | | | | | | | | | | |  |  |  |  |
|  |  | | | | | | | | Std. coef. | | *p* | 95% CI | | |  |  |  |  |
|  |  | | | | | | | |  | |  | lower | upper | |  |  |  |  |
| **Direct effects** |  | | | | | | | |  | |  |  |  | |  |  |  |  |
|  | Age → SROUTINE | | | | | | | | .017 | | *.544* | -.039 | .073 | |  |  |  |  |
|  | Gender → SROUTINE | | | | | | | | .012 | | *.571* | -.028 | .052 | |  |  |  |  |
|  | Relationship status | | | | | | | | -.006 | | *.772* | -.046 | .034 | |  |  |  |  |
|  | Program of study → SROUTINE | | | | | | | | .039 | | *.080* | -.005 | .084 | |  |  |  |  |
|  | Mode of study → SROUTINE | | | | | | | | -.013 | | *.583* | -.059 | .033 | |  |  |  |  |
|  | Offline course load → SROUTINE | | | | | | | | .015 | | *.455* | -.024 | .054 | |  |  |  |  |
|  | Online study → SROUTINE | | | | | | | | .001 | | *.956* | -.038 | .040 | |  |  |  |  |
|  | Personal study time → SROUTINE | | | | | | | | -.082 | | *<.001* | -.124 | -.039 | |  |  |  |  |
|  | Time spent in paid job(s) → SROUTINE | | | | | | | | .033 | | *.132* | -.010 | .075 | |  |  |  |  |
|  | Decrease in social contact due to COVID-19 measures → SROUTINE | | | | | | | | -.028 | | *.156* | -.067 | .011 | |  |  |  |  |
| N = 2,488 |  | | | | | | | |  | |  | |  |  |  |  |  |  |

| **Table SM3. Standardized effects for predictors in the structural model determining life satisfaction among Czech students** | | | | | |
| --- | --- | --- | --- | --- | --- |
|  |  | Std. coef. | *p* | 95% CI | |
|  |  |  |  | lower | upper |
| **Direct effects** | |  |  |  |  |
|  | Age → SLEQUAL | .063 | *.023* | .009 | .117 |
|  | Gender → SLEQUAL | .014 | *.505* | -.026 | .117 |
|  | Relationship status → SLEQUAL | -.028 | *.167* | -.068 | .012 |
|  | Program of study → SLEQUAL | .064 | *.004* | .021 | .108 |
|  | Mode of study → SLEQUAL | .041 | *.087* | -.006 | .087 |
|  | Sleep patterns → SLEQUAL | .148 | *<.001* | .108 | .188 |
| **Indirect effects** |  |  |  |  |  |
|  | Offline course load → SROUTINE → SLEQUAL | .002 | *.457* | -.004 | .008 |
|  | Online study → SROUTINE → SLEQUAL | .000 | *.956* | -.006 | .006 |
|  | Personal study time → SROUTINE → SLEQUAL | -.012 | *.001* | -.019 | -.005 |
|  | Time spent in paid job(s) → SROUTINE → SLEQUAL | .005 | *.136* | -.002 | .011 |
|  | Decrease in social contact due to COVID-19 measures → SROUTINE → SLEQUAL | -.004 | *.153* | -.010 | .002 |

N = 2,488
